# Mapping Respiratory Health Digital Interventions in South and Southeast Asia: Protocol for a Scoping Review

**DOI:** 10.2196/52517

**Published:** 2024-01-12

**Authors:** Laura Evans, Jay Evans, Monica Fletcher, Adina Abdullah, Zakiuddin Ahmed

**Affiliations:** 1 Usher Institute University of Edinburgh Edinburgh United Kingdom; 2 Department of Primary Care Medicine Faculty of Medicine Universiti Malaya Kuala Lumpur Malaysia; 3 Riphah Institute of Healthcare Improvement and Safety Islamabad Pakistan

**Keywords:** digital health, respiratory health, Asia, scoping review, landscape mapping, digital health intervention, digital health environment, mobile health, mHealth

## Abstract

**Background:**

The last 2 decades have been a time of exponential growth and maturation for digital health, while the global burden of respiratory disease continues to grow worldwide. Leveraging digital health interventions (DHIs) to manage and mitigate respiratory disease and its adverse health effects presents itself as an obvious path forward.

**Objective:**

We aimed to understand the current digital landscape and enabling environment around respiratory health to reduce costs, avoid duplication, and understand the comprehensiveness of DHIs.

**Methods:**

This study will follow a scoping review methodology as outlined by Arksey and O’Malley, the Joanna Briggs Institute, and the PRISMA-ScR (Preferred Reporting Items for Systematic Reviews and Meta-Analyses extension for Scoping Reviews) checklist. MEDLINE, Embase, CINAHL, PsycINFO, Cochrane Library, Web of Science, PakiMedNet, and MyMedR databases will be searched along with key websites, repositories, and gray literature databases. The terms “respiratory health,” “digital health,” “South Asia,” and “Southeast Asia,” as well as related terms will be searched. The results will be screened for duplicates and then against the inclusion and exclusion criteria. For the studies included, data will be extracted, collated, and analyzed.

**Results:**

The scoping review was started in July 2023 and will be finalized by February 2024. Results will be presented following the World Health Organization’s classification of DHIs to categorize interventions in a standardized format and the mobile health evidence reporting and assessment checklist to report on the effectiveness of interventions. Further exposition of the evidence extracted will be presented through narrative synthesis.

**Conclusions:**

As DHIs continue to proliferate, the need to understand the current landscape becomes more pertinent. In this scoping review, we will seek to more clearly understand what digital health tools and technologies are being used in the current landscape of digital health in South and Southeast Asia for respiratory health and to what extent they are addressing the respiratory health needs of the region. The results will inform recommendations on digital health tools for respiratory health in South and Southeast Asia will help funders and implementers of DHIs leverage existing technologies and accelerate innovations that address documented gaps in the studied countries.

**International Registered Report Identifier (IRRID):**

DERR1-10.2196/52517

## Introduction

Digital health and care refer to the use of information communication technologies, commonly known as digital health interventions (DHIs), by health and care professionals, patients, carers, or health managers to manage illnesses and wellness [1-4]. The last 2 decades have been a time of exponential growth and maturation for digital health [4] due to the promises of improved health for all and personal health empowerment [5]. Concurrently, the global burden of respiratory disease continues to grow worldwide, with infectious and noncommunicable respiratory diseases being among the top 10 medical conditions (out of 369 diseases and injuries measured) in terms of years of life lost due to premature death and years lived with a disability (measured by disability-adjusted life years) [6,7]. This increased burden of respiratory disease is more acutely felt in Asia, where mortality rates are higher and public awareness and government engagement are lower than in other regions of the world [8,9]. Leveraging DHIs to manage and mitigate respiratory disease and its adverse health effects presents itself as an obvious path forward. However, the first step to harnessing the power of digital health must be understanding the current digital landscape and enabling the environment to reduce costs, avoid duplication, and increase the efficiency, accessibility, and sustainability of such interventions [10-13].

The National Institute for Health Research (NIHR)–funded Global Health Research Unit on Respiratory Health (RESPIRE) is a collaboration between several Asian organizations and universities in Bangladesh, Bhutan, India, Malaysia, Pakistan, Indonesia, and Sri Lanka and the University of Edinburgh in Scotland, United Kingdom [9,14,15]. RESPIRE aims to achieve the following:

develop into a world-leading Unit that will: (i) map and collate continuing and emerging respiratory challenges; (ii) prioritise existing evidence-based interventions that have the potential to be adapted to reduce mortality and morbidity in low- and middle-income countries (LMICs); (iii) support local adaptation and tailoring of interventions for deployment in low-resource environments and catalyse developmental work in areas of unmet need; (iv) support local implementation efforts and evaluation of programmes of work; and (v) identify the best delivery mechanisms for long-term delivery and scaling-up.

This is done through 4 different translational platforms, of which 1 platform focuses on Digital Health and Innovation and aims to provide holistic support to partner countries on the design, funding, deployment, and sustainability of new and existing digital health interventions for respiratory health. This scoping review will contribute to advancing the aims and work of Digital Health and Innovations.

Understanding the current landscape of DHIs that target respiratory diseases will (1) uncover existing gaps, (2) highlight potential opportunities, (3) suggest research and program priorities most needed in the field of digital health to address current respiratory health diseases in South and Southeast Asia, and (4) further advance RESPIRE’s overall aims. Therefore, we aim to undertake a scoping review to map respiratory disease DHIs in South and Southeast Asia to identify existing technologies, opportunities, and gaps, and to put forward pertinent recommendations from the insights gained.

## Methods

### Scoping Review Methodology

The scoping review methodology, as outlined by Arksey and O’Malley and the Joanna Briggs Institute (JBI) [16,17], is an appropriate approach for mapping DHIs. Scoping reviews allow flexibility when exploring the diverse and heterogenous field of digital health, are appropriate when using different sources of data (eg, peer-reviewed journals, gray literature, and expert opinions), and permit inclusion and exclusion criteria to be iteratively refined as more evidence is uncovered [16-19]. Additionally, the PRISMA-ScR (Preferred Reporting Items for Systematic Reviews and Meta-Analyses Extension for Scoping Reviews) [20] will be followed to ensure adherence to methodological standards.

### Identifying the Research Question

This scoping review will seek to answer the following research questions:

1. What digital health tools and technologies are being used in South and Southeast Asia for respiratory health?
2. How are these addressing (or not) the respiratory health needs of the region?
3. What recommendations can be made from the literature?

### Identifying Relevant Studies

To identify relevant literature, academic databases will be searched along with key websites, repositories, and gray literature databases that may contain relevant information for our scoping review. Textbox 1 shows the proposed databases.

The search strategy has been initially drafted for MEDLINE in the Ovid platform (Textbox 2) and will be adapted for the remaining databases. The search strategy has been iteratively developed and refined by the authors’ input and the librarian at the University of Edinburgh. The terms “respiratory health,” “digital health,” “South Asia,” and “Southeast Asia,” as well as all relevant variations of these terms have been included in the search strategy to gather as much pertinent literature and information as possible.

**Proposed database and key websites.**

**Databases**

- MEDLINE
- Embase
- CINAHL
- PsycINFO
- Cochrane Library
- Web of Science
- PakMediNet
- MyMedR

**Other sources**

- ProQuest Thesis and Dissertations
- Digital Health Atlas
- Global Digital Health Index
- Digital Square’s Map and Match
- United Nations Children’s Emergency Fund’s Mapping of Digital Health Tools and Technologies
- World Health Organization’s Global Index Medicus

**Search strategy.**

**Search terms**

1. occupational diseases/ or Asthma/ or Air Pollution/ or Respiratory Tract Diseases/ or Occupational Exposure/ or Air Pollutants/ or Tuberculosis/ or Tuberculosis, Pulmonary/ or “Tobacco Use”/ or Tobacco/ or “Tobacco Use Cessation”/ or “Tobacco Use Cessation Devices”/ or Pulmonary Disease, Chronic Obstructive/ or Pneumonia/ or COVID-19/
2. (respiratory health or tuberculosis or tobacco or pneumonia).mp
3. 1 or 2
4. telemedicine/ or telehealth/ or artificial intelligence/ or machine learning/ or medical informatics/ or electronic health records/ or mobile applications/ or exp Informatics/
5. (artificial intelligence or digital health or e-health or ehealth or m-health or mhealth or (digital adj2 (health or solution* or system*)) or (health adj2 (electronic or record* or tele*)) or ict4d or (information adj5 development) or machine learning or mobile health or telecare or telehealth or telemedicine or tele-health or teleconsultation or tele-consultation or tele-care or tele-medicine or (tele adj1 (medicine or care or health or consultation)) or ((virtual* or remote*) adj4 (visit* or consult* or meet* or appoint* or communicat*)) or (Health* adj4 tech*) or e-portal* or eportal* or (Patient* adj2 portal*) or (medical adj2 informatic*)).mp.
6. 4 or 5
7. Asia/ or Asia, Southern/ or Asia, Southeastern/
8. (Asia or Brunei Darussalam or Cambodia or Indonesia or Lao People’s Democratic Republic or Malaysia or Myanmar or Philippines or Singapore or Thailand or Timor-Leste or Viet Nam or Vietnam or Afghanistan or Bangladesh or Bhutan or India or Iran or Islamic Republic of Iran or Maldives or Nepal or Pakistan or Sri Lanka).mp
9. 7 or 8
10. 3, 6, and 9

### Study Selection

Exclusion and inclusion criteria have been developed according to the domains of population, concept, context, and type of evidence, suggested by the JBI [17,18]. Additionally, an “other variables” category has been created to include year, language, and format criteria (Table 1). The regions of South and Southeast Asia include 19 countries as established by the United Nations Statistics Division [21]. These 2 regions have been chosen because they contain all the RESPIRE2 countries. Multicountry studies containing countries from the selected regions and other regions of the world will be included in the initial screening and only excluded if they do not provide the data of interest separate for each country. Only studies in English and those published in the last 10 years (since 2013) will be included to keep the scope of this review within manageable boundaries.

**Table 1:** Inclusion and exclusion criteria.

Domain	Inclusion criteria	Exclusion criteria
Population	Any population	N/A^a^
Concept	Technological interventions for respiratory health that fall under any of the categories of the World Health Organization’s classification of digital health interventions Respiratory health includes respiratory infections, noncommunicable respiratory diseases, and preventable risk factors for respiratory conditions, as defined by RESPIRE^b^	Other nontechnological interventions used for respiratory health Not a respiratory health focus
Context	South and Southeast Asia	The rest of the world
Types of evidence sources	Any study design	Books Abstracts only Posters Protocols
Other variables	Published in English Studies or data published in the last 10 years (2013-2023) Full article or data are available digitally	Published in any other language Studies or data published before 2013 Full article or data are not available digitally

^a^N/A: not applicable.

^b^RESPIRE: The National Institute for Health Research–funded Global Health Research Unit on Respiratory Health.

For information found in a scientific study format, Covidence software [22] will be used to eliminate duplicates and carry out screening and extraction. After deduplication, title, abstract, and full-text screening will be done by 2 authors according to the inclusion and exclusion criteria. Discrepancies will be first addressed by consensus between those 2 authors. If there is a lack of consensus, a third reviewer will decide.

For all other types of information or data found from searches, manual screening by 1 reviewer will happen first. Relevant data will be entered into a spreadsheet, and a second reviewer will assess it. Discrepancies will first be addressed between both reviewers, and if there is no consensus, a third reviewer will make the final decision. We will not contact authors directly to further understand whether a study should be included since it would most likely significantly lengthen the timeline for this scoping review.

Scoping reviews use secondary data and do not require ethics approval under RESPIRE rules. However, the authors will adhere to the highest ethical standards when carrying out the review. This protocol establishes a transparent and reproducible study design, which limits the potential for personal bias [23].

### Charting the Data

After selection, relevant data will be extracted to a spreadsheet using Covidence. The extraction form will be first piloted in 3-5 studies to assess if it is fit for purpose as recommended by the JBI. Data selected from a nonscientific study format will be entered into the extraction spreadsheet as accurately as possible. However, it is acknowledged that not all fields may contain relevant information, and some fields may need to be modified (eg, data from the Digital Health Atlas will have a “source of information” field instead of an “author” field).

When 2 or more articles refer to the same overall study, those articles will be grouped as one before data extraction.

### Collating, Summarizing, and Reporting the Results

After data analysis, the data will be collated and analyzed as follows:

- Quantitative data (ie, the number of studies, type of study, and year) will be presented.
- Qualitative data will be presented following the World Health Organization’s classification of digital health interventions [24] to categorize interventions in a standardized format and the mobile health evidence reporting and assessment checklist [25] to report on the comprehensiveness of the interventions.
- Narrative synthesis will be used to answer the research questions and to present further data extracted.

### Consultation

Consultation with stakeholders will be ongoing throughout the scoping review process. We will disseminate early findings among partners so that they can provide feedback on findings and that feedback can be incorporated into the discussion.

## Results

This scoping review was started in July 2023 and will be finalized by February 2024. Preliminary findings will be shared with stakeholders and a final write-up of the scoping review is projected to be finalized by the end of March 2024. To date, 10,469 studies have been screened. The screening of abstracts is underway.

## Discussion

### Expected Outcomes

Through this scoping review, we will seek to better understand what digital health tools and technologies are being used in South and Southeast Asia for respiratory health and to what extent they are addressing the respiratory health needs of the region. The results will inform recommendations on digital health tools for respiratory health in South and Southeast Asia and will help funders and implementers of DHIs leverage existing technologies and accelerate innovations that address documented gaps in the studied countries. The results of this review will be limited by the fact that only studies in English and studies published in the last 10 years will be included. This review will enhance the knowledge of digital health tools and technologies in the region, which is paramount before undertaking any new initiative, as it helps prevent redundant work and investment by building on existing systems and lessons learned.

### Conclusions

As DHIs, in general and in respiratory health in particular, continue to proliferate, the need to understand the current landscape becomes more pertinent. Through this scoping review, we will systematically map out DHIs, which serves as the required first step in any well-informed and thought-out design and deployment of DHIs.

## Appendix

Multimedia Appendix 1 Peer-review reports from the National Institute for Health and Care Research (NIHR).

## Abbreviations

DHI: digital health intervention
JBI: Joanna Briggs Institute
NIHR: National Institute for Health Research
PRISMA-ScR: Preferred Reporting Items for Systematic Reviews and Meta-Analyses Extension for Scoping Reviews
RESPIRE: National Institute for Health Research–funded Global Health Research Unit on Respiratory Health

## Data Availability

The data resulting from this scoping review will be made available as supplementary materials at the time of publication.
