# Supplementary material for: Mapping Respiratory Health Digital Interventions in South and Southeast Asia: Protocol for a Scoping Review
Source: JMIR Res Protoc. 2024 Jan 12;13:e52517. doi: 10.2196/52517 (PMC10818234; doi:10.2196/52517)
Supplement: Multimedia Appendix 1 [file resprot_v13i1e52517_app1.pdf]

NIHR Global Health Research Unit on Respiratory Health (RESPIRE)
The University of Edinburgh,
Medical School, Teviot Place,
Edinburgh
EH8 9AG

Review
Mapping respiratory health digital interventions in Bangladesh, Bhutan, India, Malaysia, Pakistan, Indonesia, and Sri Lanka: Protocol for a scoping review

NIHR Global Health Research Unit on Respiratory Health (RESPIRE)
Funded by the National Institute for Health and Care Research (NIHR), RESPIRE is a Global Health Research Unit focusing on respiratory health in Asia. It is led from the University of Edinburgh. Digital Health and Innovation is a supporting platform that provides and advise on digital health and innovation to the three clinical programmes on Non-Communicable Diseases (NCDs), infectious diseases and risk factor prevention.

RESPIRE comprises a broad portfolio of projects of which this scoping review was one. The over-arching RESPIRE proposal, which included this proposed mapping of Respiratory Health Digital Interventions in Bangladesh, Bhutan, India, Malaysia, Pakistan, Indonesia, and Sri Lanka, was reviewed by the funder who commented on the '*mobile phone-based warning systems for CRD'* highlighting the need for both quantitative and qualitative evidence as is proposed in this scoping review. The team for this application includes personnel from the central Edinburgh team (JE, KLE and MF) as well as colleagues from Malaysia (AA) and Pakistan (ZA) and will systematically map respiratory digital health interventions from all the RESPIRE partner countries (Bangladesh, Bhutan, India, Malaysia, Pakistan, Indonesia, and Sri Lanka). This broad approach aligns with a critical comment from an NIHR appointed peer reviewer who stated: *'Although Malaysia is a good location in which to test the digital health resource and e-learning platform for RESPIRE-2, in many respects it does not represent the relatively deprived and more typical communities in many LMICs'*

The budget is entrusted to senior RESPIRE leadership to review and shape detailed proposals for each of the approved projects. I am thus reviewing this in my capacity as co-lead of the RESPIRE NCD programme of work.

Thank you for responding to my comments. In general, I am happy with your revisions. If I have any additional comments, I have added these in red.

Title

- Not essential, but I think it would be good to include the word 'systematic' in the title. It clarifies that this a systematic process of searching and data extraction.
    - Response: Including the word 'systematic' may be confusing since we are intending to do a scoping review and not a systematic review.

The University of Edinburgh is a charitable body, registered in Scotland, with registration number SC005336.

- Entirely up to you! My thought was that you might want to describe it as a 'systematic scoping review' as my experience is that some readers/editors do not appreciate that a 'scoping review' is a systematic process. I think there is confusion with the colloquial use of initial 'scoping' when developing search strategies for systematic reviews.
  - Response: Thank you for the clarification. I understand the concern, however we think that keeping it as 'scoping review' is more in line with good methodology even if some readers misunderstand the meaning of 'scoping' in this context.

Abstract

- I appreciate the difficulty of including all details in a word-limited abstract, but I would like to see reference to methodology such as: high level search terms; duplicate selection and data extraction processes; and analytical approach (not just 'will be analysed'!). Mention of the six steps of scoping review methodology would be helpful.
  - Response: this has been included
- The objective (To understand the current digital landscape and enabling environment around respiratory health) is entirely appropriate for a scoping review, but this is not reflected in the statement of results which states 'effectiveness of interventions' will be reported. This is not normally appropriate in a scoping review in which quality of included studies is not formally assessed and grading of the conclusions will not be possible.
  - Response: This has been changed, see below a response regarding 'effectivness'
- Unless the journal insists on the abstract headings (Background; Objective; Methods; Results; Conclusions), I suggest increasing the space given to 'Methods', delete the heading for 'Results' (because you haven't got any yet) and change the heading 'Conclusions' to 'Discussion' (where your existing two sentences will be fine!).
  - Response: the headings are established by the journal on both the abstract and the main body (including the section on results) but the word count for the abstract is higher than initially thought, so we can iclude the suggestions above.
- Add 'systematic scoping review' to the key words
  - Response: added.
  - Thank you. You don't need both 'scoping review' and 'systematic scoping review'. My preference is for the latter as explained above. (Environment' is misspelt)
    - Response: changed the spelling of environment. Also, deleted 'systematic scoping review'.

Introduction

- The rationale is well stated and the aim (to map respiratory digital health interventions in Bangladesh, Bhutan, India, Malaysia, Pakistan, Indonesia, and Sri Lanka to identify existing technologies, opportunities, and gaps) is entirely appropriate for a scoping review.
  - Response: scope changed to South and Southeast Asian countries. See below.
- P3; para 1: 'non-infectious respiratory disease' might be better as 'non-communicable diseases' which is the WHO preferred term.
  - Response: changed

Methods

You correctly plan to follow the six steps of Arksey and O'Malley framework. It would be clearer if you then number/name the sub-sections accordingly so that the reader understands how you plan to fulfil each step:

1. *Identifying the research question*

You have a clear aim, but it might be helpful to break this down into objectives (or research questions) as that will help you clarify selection criteria, define data extraction and undertake analysis (and will help you avoid 'creep' such as mentioning effectiveness…)

- Response: Research questions added

2. *Identifying relevant studies*

The search strategy is described. Please justify why only papers in English? We have partners in all the RESPIRE countries who could translate? The start date of 2013 is probably reasonable in a fast-moving field such as technology, but it would be good practice to justify.

- Response: We are including studies only in English because after a brief consultation with some RESPIRE2 partners, it doesn't appear feasible form a time perspecitve to include other languages. 2013 was chosen to look at the last 10 years of digital health interventions, because the field really does change fast.
- OK – but restricting to English this will need to be highlighted as a limitation
    - Response: Agreed, we will highlight this in the limitations section.

3. *Study selection*

What software will you use? Will this be duplicate screening? How will you train the researchers? What standards of agreement do you regard as acceptable? How will you resolve disagreements? What will be the process for making decisions on how to operationalise the inclusion/exclusion criteria?

- Response: these questions were addressed under the 'charting the data' section. Nnow they have been moved to 'study selection' and revised slightly to be more clear.

Table 3.

- In column 2 you state 'respiratory infections (pneumonia and tuberculosis)'. Are you restricting to these two conditions and excluding conditions such as RSV, COVID), or should this be '(e.g. pneumonia and tuberculosis)'?
    - Response: changed to 'respiratory infections' for a broader scope
- Similarly with 'non-communicable respiratory diseases (asthma and chronic obstructive pulmonary disease [COPD])' and preventable risk factors for respiratory conditions (tobacco smoking and exposure to air pollution). I presume they are exemplars.
    - Response: changed to 'non-communicable respiratory diseases' for a broader scope
- What counts as 'technology for respiratory health'. Telephone calls? Text messages? Is there anything that is typically counted as technology that you wish to exclude?
    - Response: nothing in particular that we wish to exclude. the search terminology is very broad to include as many forms of technology as possible. However, we should offer a clear definition of digital health in the scoping review manuscript, so that it is clear what is guiding our decision-making process
    - I strongly suggest you define boundaries in the protocol or you will not know whether to include/exclude the studies you find. Some technology is obvious, but there are grey areas (e.g. telephone calls without any other technology). You will need to 'operationalise' the inclusion/exclusion during the review process, but you need to set clear boundaries in the protocol.
        - Thank you for further explanation. I agree the there are many grey areas. We have modified the inclusion criteria to read 'Technological interventions for respiratory health that fall under any of the categories of the World Health Organization's (WHO) classification of digital health interventions.' As a way to provide more boundaries, but still keeping it broad.
- You have included 'Bangladesh, Bhutan, India, Malaysia, Pakistan, Indonesia, and Sri Lanka' but excluded 'Countries outside of Asia'. What about a study in Nepal? Myanmar – or any other Asian country that is not the seven RESPIRE partners.

- Response: We understand that this can seem arbitrary, therefore to undertake a more relevant scoping review that still is useful for RESPIRE work and partners, we have broaden the region to South and Southeast Asia (as defined by the UN).
- A good decision!

- What will you do with papers from several countries – say Bhutan that would be included and Nepal that would not?
    - Response: the previous response addresses this issue.
    - Yes, but the principle remains. What if a study reported findings from a South/Southeast Asian country and (say) and an African LMIC? You need a rule about what you will do if only part of the data is relevant to your inclusion criteria. Typically, reviewers state that they will include such studies if the data of interest are presented separately (e.g. as sub-groups in tables) or if the population of interest forms the majority of the total population (for example if combined data were from eight South/Southeast Asian countries and one African country). My thought would be that the former should be included, and the latter probably excluded.
        - Response: this is a very good observation. It has been changed in the manuscript to reflect that if relevant data is not provided separate for each country, the study will be excluded.
- Will you contact authors if it is not clear if a paper should be included
    - Response: Although we acknowledge that that would be ideal, we will not as it would draw out the process to long.
    - I may have missed it, but I can't see where you have added this.
        - Response: This has now been adeed.
- What will you do with abstracts. In column 3 you say 'exclude' – but will you use it to try and find a published paper?
    - Response: We have changed to 'abstracts only'. If just an abstract was published (for example during a conference preceding) those will be excluded, but if an abstract belongs to a published paper and we find such paper, then it would be included.
- What will you do if you find a study protocol?
    - Response: study protocols will be excluded as it is likely that they will not provide relevant information on technological tools used. However, if a study protocol appears relevant, we will search to see if the actual study has been done and results published.

4. *Charting the data*

The duplicate data extraction process is described.

- Will you contact authors to clarify details?
    - Response: Although contacting authors for clarification would be the best approach, we don't think it would be feasible due to a our restricted timeline, so we will not contact them.
    - Again, this needs to be stated in the protocol text
        - This has now been added
- If you find two papers reporting one study will you link them as one study?
    - Response: Yes, we will link them and treat them as one study

5. *Collating, summarising and reporting the results*

The description of using the WHO classification of digital health interventions and the mobile health (mHealth) evidence reporting and assessment (mERA) checklist is appropriate but should be in step 5 of the scoping review steps ('Results' does not seem a relevant category for a protocol as results is one thing that you do not have!!). My main concern is the use of the word 'effective' as a scoping review it not an appropriate methodology for estimating 'effectiveness'.

This is where you can pick up on your objectives and explain how you will analyse your data to answer the focused research questions you defined in step 1. Typically in scoping reviews analysis is descriptive, or involve developing matrices, or figures to illustrate the scope of the innovations that you identify.
There is no mention of how you will synthesise qualitative data (which I presume you are including as your table states 'any study design)

- Response: the word 'efectiveness' first came from the mERA checklist, but we agree that it is outside of the scoping review methodology to assess such effectiveness. The word 'effectivenes' has now be changed to 'comprehensiveness' of an intervention.
- Response: we have added now how we will sumarrise and present the data.

This description of the analysis is improved, but the heading to your three bullets should read 'The data will be collated and analysed as follows: xxxxx
Then you can state that the findings will be summarised and presented to answer the three research questions that you have defined (1. What digital health tools and technologies are being employed in South and Southeast Asia for respiratory health? 2. How are these addressing (or not) the respiratory health needs of the region? 3. What recommendations can be made from the literature?). This might be good text to put in the 'Results' section!
Response: a line has been added to included that the narrative section will be used to answer the research questions and to further discuss other data extracted.

6. *Consultation*
RESPIRE is an ideal collaboration to enable a consultation phase in which you can feedback the findings and learn from the feedback.

- Response: this section has been added.

Typos and grammatical points:

- P4; 'Scoping review methodology: '… is an appropriate approach for mapping digital health interventions'
    - Response: this has been changed

Patient and Public Involvement
I think you need to explain why you do not have any patients involved… Maybe better to broaden this to Community Engagement and Involvement and include any related stakeholder work rather than just patients.

- This section has been removed and information on stakeholder engagement has been added to the 'consultations' section

Discussion
The discussion is appropriate.

Hilary Pinnock
Professor of Primary Care Respiratory Medicine, The University of Edinburgh.
• NIHR Global Health Research Unit on Respiratory Health (RESPIRE)
29th June 2023

THE UNIVERSITY of EDINBURGH
Global Health Academy

Director Professor Liz Grant
Global Health Academy

Centre for Population Health Sciences
Usher Institute
Teviot Place
Edinburgh, EH8 9AG
www.globalhealthacademy.ed.ac.uk
Email globalhealth@ed.ac.uk
Phone +44 131 651 4138

Paper: Mapping respiratory health digital interventions in Bangladesh, Bhutan, India, Malaysia, Pakistan, Indonesia, and Sri Lanka: Protocol for a scoping review

Review:
This is an important piece of research which will add to the literature and provide a valuable springboard for others.
Important to complete now with the rapid increase of respiratory health interventions
It is well written and concise –
There are a few changes that will help the paper – especially more detail on the processes that you will adopt to carry out the various parts – screening, mediation and resolution, and synthesise of all the data
Some comments:

- Worth condensing the methods and add in the inclusion exclusion criteria (or at least an indication of) and the search terms
  - Response: the methods section has been restructured according to the other reviewer's comments. Inclusion and exclusion criteria and search strategy is included as suggested.
- Good that you flagged up the categorization process according to WHO DH interventions though put this in the methods section – as the results are what you will find when completing the review
  - Response: This has been moved to Methods>Collating, summarising and reporting the results as suggested
- Are your keywords complete – should you include digital health environment and scoping review
  - Response: Those are no added
- In this sentence I would put non-communicable respiratory diseases *Concurrently, the global burden of respiratory disease continues to grow worldwide, with infectious and non-infectious respiratory diseases being among the top ten medical conditions (out of 369 diseases*
  - Response: this has been changed to non-communicable respiratory diseases
- In the paragraph on RESPIRE the last sentence is a little clumsy and could be rephrased *It is under this platform that this proposed scoping review will be done.*
  - Response: this has now been changed to: " This scoping review will be done to help further the 'Digital Health & Innovations' aims and work."

- Worth saying you are using the updated Arksey and O'Malley framework (and I don't think you are doing step 6 of A &O'M)
    - Response: Is step 6 the consultation process? If so is now added.
- Can you justify your dates – why 2013?
    - Response: this is now in the text: "Only studies in English and those published in the last ten years (since 2013) will be included to keep the scope of this review within manageable boundaries."
- Do you have a sense of the non English literature – if it isn't much it may be worth including.
    - Response: This is now justified in the text: "Only studies in English and those published in the last ten years (since 2013) will be included to keep the scope of this review within manageable boundaries.". We have consulted with partners in Pakistan and Malaysia, and both recommended keeping results to English as they didn't think that anyone in their team had the capacity to go through local language results at the moment.
- how are you managing the COVID literature recognizing the surge in literature at this point and the shift in ways of digital use which may revert back
    - Response: COVID has now been included in the search strategy, although during the screening process, for a paper to be included, it would need to be clear that the authors were focusing on the respiratory aspects of COVID (instead of other aspects such as long COVID). Since this a scoping review and affords some flexibility in the methodology as results are uncovered, if results containing COVID-related interventions are disproportionally more than the rest of diseases, we may choose to present those results by themselves so that clear insights can also be gathered from the non-COVID interventions.
- Do you need to match up your search strategy and your inclusion/exclusion box? There looks like a few discrepancies.
    - Response: yes, indeed. That has been corrected now.
- With the excluded countries would it be better to say all other countries not listed ? Though what will you do if papers are from a mix of countries – some from your included countries and others outside it. And if more than one paper is reporting the same study how will you capture that You note in the discussion that you will understand the digital landscape of 7 countries chosen and to some extent other Asian countries .
    - Response: We have changed the country list to include all the UN countries in South and Southeast Asia since they contain all the RESPIRE2 countries, and the results may be more relevant for the scientific community beyond respire.
    - Multi-countries studies will be included and only excluded if they fail to meet the other exclusion criteria. This is now in the text of the protocol.
- You say that you will report on the effectiveness of the interventions but our objective is to understand what creates the enabling environment with 5 criteria – on cost, efficiency, redundancy, access and sustainability
    - Response: the language around effectiveness has now been eliminated since this is a scoping review, per other reviewer suggestions. the mERA checklist covers those 5 criteria and several others.
- With Patient and Public Involvement it would be useful to add a further line in this - and I would have anticipated that you would have some public involvement in this – with the breadth of the scoping review checking grey lit. Will you seek advice from patient champions on grey lit? Or should you include Step 6?
    - Response: The patient and public involvement section has now been replaced by the consultation section. We will consult with partners across RESPIRE2 countries. A wider consultation would undoubtedly be helpful but not possible from a time and human resources point of view.

These are all very short suggestions on clarification and explanation.

Liz Grant

Professor Liz Grant
Assistant Principal Global Health and
Director, Global Health Academy

RESPIRE KNOWLEDGE TRANSLATION HUB LEAD
NIHR Global Health Research Unit on Respiratory Health (RESPIRE)
